# From Food Waste to Innovative Biomaterial: Sea Urchin-Derived Collagen for Applications in Skin Regenerative Medicine

**DOI:** 10.3390/md18080414

**Published:** 2020-08-06

**Authors:** Cinzia Ferrario, Francesco Rusconi, Albana Pulaj, Raffaella Macchi, Paolo Landini, Moira Paroni, Graziano Colombo, Tiziana Martinello, Luca Melotti, Chiara Gomiero, M. Daniela Candia Carnevali, Francesco Bonasoro, Marco Patruno, Michela Sugni

**Affiliations:** 1 Department of Environmental Science and Policy, University of Milan, Via Celoria, 2, 20133 Milan, Italy; franci.ruska@gmail.com (F.R.); pulaj44@gmail.com (A.P.); daniela.candia@unimi.it (M.D.C.C.); francesco.bonasoro@unimi.it (F.B.); 2 Center for Complexity and Biosystems, Department of Physics, University of Milan, Via Celoria, 16, 20133 Milan, Italy; 3 Department of Biosciences, University of Milan, Via Celoria, 26, 20133 Milan, Italy; raffaella.macchi@unimi.it (R.M.); paolo.landini@unimi.it (P.L.); moira.paroni@unimi.it (M.P.); graziano.colombo@unimi.it (G.C.); 4 Department of Veterinary Medicine, University of Bari, SP. Casamassima Km.3, 70010 Valenzano (Ba), Italy; tiziana.martinello@gmail.com; 5 Department of Comparative Biomedicine and Food Science, University of Padua, Agripolis Viale dell’Università, 16, 35020 Legnaro, Italy; luca.melotti.4@phd.unipd.it (L.M.); chiara.gomiero@phd.unipd.it (C.G.); 6 GAIA 2050 Center, Department of Environmental Science and Policy, University of Milan, Via Celoria, 2, 20133 Milan, Italy

**Keywords:** fibrillar collagen, sea urchins, marine collagen-based skin-like scaffolds, eco-friendly biomaterial, regenerative medicine

## Abstract

Collagen-based skin-like scaffolds (CBSS) are promising alternatives to skin grafts to repair wounds and injuries. In this work, we propose that the common marine invertebrate sea urchin represents a promising and eco-friendly source of native collagen to develop innovative CBSS for skin injury treatment. Sea urchin food waste after gonad removal was here used to extract fibrillar glycosaminoglycan (GAG)-rich collagen to produce bilayer (2D + 3D) CBSS. Microstructure, mechanical stability, permeability to water and proteins, ability to exclude bacteria and act as scaffolding for fibroblasts were evaluated. Our data show that the thin and dense 2D collagen membrane strongly reduces water evaporation (less than 5% of water passes through the membrane after 7 days) and protein diffusion (less than 2% of BSA passes after 7 days), and acts as a barrier against bacterial infiltration (more than 99% of the different tested bacterial species is retained by the 2D collagen membrane up to 48 h), thus functionally mimicking the epidermal layer. The thick sponge-like 3D collagen scaffold, structurally and functionally resembling the dermal layer, is mechanically stable in wet conditions, biocompatible in vitro (seeded fibroblasts are viable and proliferate), and efficiently acts as a scaffold for fibroblast infiltration. Thus, thanks to their chemical and biological properties, CBSS derived from sea urchins might represent a promising, eco-friendly, and economically sustainable biomaterial for tissue regenerative medicine.

## 1. Introduction

Skin injuries, such as chronic wounds, ulcers and burns, are one of the most common, and socially and economically relevant, problems in human health care, with an estimated cost of more than US $25 billion per year in the United States alone [1]. Nowadays, various kinds of skin injuries can be partially repaired using skin allografts, autografts and xenografts [2]. However, these techniques have many limitations, such as the availability of donor site, immune rejection in allogenic skin graft, slow healing, cross-infections, scarring, and high pain level for the patients [3]. Considering the complexity of skin wound healing [4], scientific research is currently aiming at finding alternatives to skin grafts to ensure effective skin healing and support regeneration of damaged tissues, while limiting their side effects [5]. The most promising tools currently under investigation in regenerative medicine are wound dressings and bioengineered skin substitutes (SS). SS are medical devices designed to reproduce the structural, mechanical and physiological features of in vivo skin [6]. Among the materials used to produce SS, collagen is arguably the most suitable since it is the main extracellular matrix (ECM) protein component and it has an important structural, functional and mechanical role in the connective tissues. Therefore, a collagen-based biomaterial can be considered the best in mimicking the ECM microenvironment [7]. Moreover, due to its biodegradability, collagen can be easily tuned/modified/substituted by cells of the neo-forming tissue and its degradation within the regenerating tissue does not lead to toxic compounds/molecules but rather helps tissue restoration since collagen peptides are considered bioactive molecules [8,9,10]. For all these reasons, collagen-based skin-like scaffolds (CBSS), such as Integra^®^ and Matriderm^®^, which are designed to temporarily or permanently close the wound, are among the most commonly used in biomedicine [11]. However, they often show drawbacks among which the absence of other ECM molecules, such as glycosaminoglycans (GAGs), that must be artificially added. Additionally, most CBSS need a two-step operation, with excessive exudate or bleeding or require subsequent removal of the external layer [12] and therefore prolonged healing times. Hence, regenerative medicine is in strong need of effective CBSS able to protect wounds from infections and fluid loss, promote tissue integration with rapid adherence and neo-vascularization, and ensure mechanical stability and durability [13].

CBSS are usually produced with collagen of bovine, porcine and equine origin (Integra^®^, Matriderm^®^, Transcyte^®^, Decutastar^®^, etc.). However, disease (i.e., BSE) transmission risks, high costs and inefficiency of recombinant technologies have commonly been observed with animal-derived CBSS. In addition, their use is also limited in some population groups due to religious and cultural beliefs. Thus, studies aimed at the identification of alternative collagen sources have been steadily increased over the last years [14,15].

Marine organisms (sponges, jellyfish, molluscs and fish) are promising sources of collagen [14,15,16,17,18,19,20,21,22]. However, most of these marine-derived collagens are used in their hydrolyzed form, a step necessary for their efficient extraction. This results in two main limitations: first, although collagen can be re-assembled in vitro to form fibrils [23], they often fail to fully reconstitute the original structure and functional efficiency, thus providing sub-optimal mechanical features [24]; secondly, associated molecules, i.e., GAGs, are generally lost during hydrolysis and need to be artificially added in order to reproduce characteristics of the ECM, such as for example hydration [25]. This is the case of the “gold standard” Integra^®^, in which a bovine-derived CBSS is artificially enriched with shark-derived chondroitin sulfate (the most common GAG of the dermis).

Echinoderms, and especially sea urchins, are the most promising source of collagen among marine invertebrates [26,27]. The advantages of using these animals are three-fold: (i) the possibility to extract collagen fibrils in their native conformation [26,27] obtaining a material similar to the in vivo microenvironment; (ii) the opportunity to naturally obtain GAG-decorated collagen fibrils [26,27,28] from mutable collagenous tissues (MCTs) [29], thus allowing the production of CBSS fully proficient in their mechanical properties; and (iii) the possibility to extract fibrillar collagen from sea urchin wastes originating from the food industry (restaurants and seafood enterprises), thus following circular economy principles and greatly reducing costs [26]. 

Indeed, in the last decades, the global demand for sea urchins has significantly increased [30]. Since the edible gonads are about 10% of the fresh animal and, therefore, the remaining about 90% is food waste, the recycling and valorization of this waste make sea urchins potential and remarkable high-value “blue by-products” to produce marine-derived biomaterials with unique features (GAG-decorated fibrillar CBSS) for tissue regeneration applications. 

Since the biomaterial employed to promote skin regeneration should mimic as much as possible the in vivo epidermis/dermis system, having a marine-derived CBSS that reproduce the ECM microenvironment from a structural, mechanical, physiological and functional point of view will be an added value for regenerative medicine.

In this work, we describe the production and characterization of a sea urchin-derived CBSS constituted by an epidermis-like layer (a thin 2D collagen membrane) and a dermis-like layer (a sponge-like 3D collagen scaffold), and deeply characterize it in terms of microstructure, mechanical stability, permeability to water, proteins and bacteria, and cell viability, proliferation and infiltration. Overall, our data indicate that collagen derived from sea urchin wastes is a promising biomaterial for biomedical applications and that “blue biotechnologies” can be the future for the sustainable management of seafood wastes in a circular economy perspective.

## 2. Results

### 2.1. Sea Urchin Fibrillar Collagen Extraction and Extraction Yield

When purified using the collagen extraction protocol described in Materials and Methods, the obtained fibrillar collagen suspension had a mean yield of 4.93% ± 2.22% (SD) of the total peristomial membrane (PM) fresh weight. The average fresh PM weight was 0.083 g ± 0.015 g (SD). Therefore, from a single PM, it was possible to obtain on average 4.5 mg of fibrillar collagen.

### 2.2. 2D Collagen Membrane Permeability Tests

**Figure 1:**
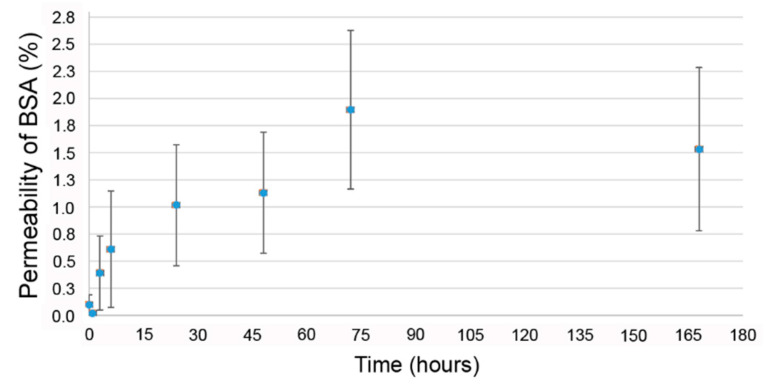
Percentage of bovine serum albumin (BSA) passing through the 2D collagen membranes. Time is expressed in hours. Mean ± standard error (SE).

2D collagen membrane permeability to both distilled water and protein was evaluated over a 7-day period, as described in Materials and Methods. Visual inspection indicated that no distilled water crossed the 2D collagen membranes at any time-point in “dry-wet” conditions (“dry” skin wound). In “wet-wet” conditions (“moist” skin wound), at 1 day the mean distilled water permeability was less than 2% (1.89% ± 0.38% (SD)). At this time-point permeability was on average 0.016 mL/cm^2^. At 7 days, the mean distilled water permeability was less than 5% (4.45% ± 0.57% (SD)). At this time-point permeability was on average 0.029 mL/cm^2^. Overall, the 2D collagen membranes could be considered a good barrier against water evaporation. For protein permeability, less than the 2% (mean value: 0.66 mg/mL) of bovine serum albumin (BSA) passed through the membrane in 7 days (Figure 1). Thus, the 2D collagen membrane could be considered almost impermeable to proteins, at least in this range of time.

### 2.3. Bacteria Infiltration Tests

**Figure 2:**
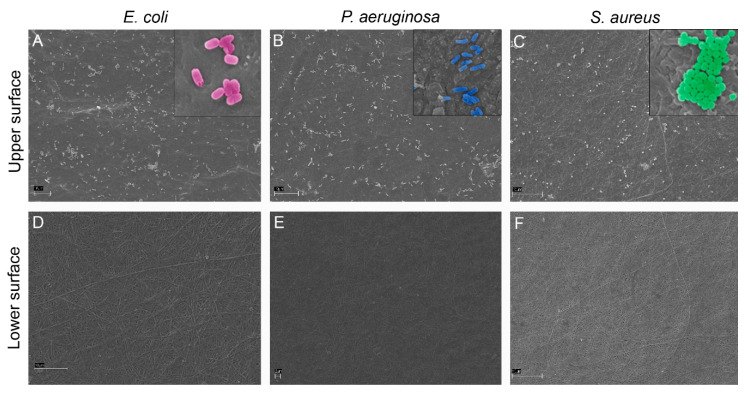
SEM micrographs of the bacteria infiltration test showing the upper and the lower surfaces of the 2D collagen membranes at 48 h. **A**: *E. coli,* upper surface. **B**: *P. aeruginosa,* upper surface. **C**: *S. aureus,* upper surface. **D**: *E. coli,* lower surface. **E**: *P. aeruginosa,* lower surface. **F**: *S. aureus,* lower surface. Bacteria are present on the upper surfaces of the 2D collagen membranes, but they are never detectable on the lower surfaces. Inserts in **A**, **B** and **C**, showing bacteria shapes, are pseudo-colored with the Software Adobe Photoshop CS3 Extended. Scale bars: **A**–**D**,**F** = 10 µm; **E** = 2 µm.

Three bacterial species characterized by different shapes (rod- or round-shaped) and dimensions (from 1 to 3 μm), were used to evaluate the proficiency of the 2D collagen membranes to prevent bacteria invasion of the skin injury. Regardless of bacteria shape and dimension, overall less than 0.117% of the initial bacteria concentration (5 × 10^6^/mL) passed through the 2D collagen membranes (Table 1). SEM analysis of the upper surfaces of the 2D collagen membranes at 48 h confirmed this result (Figure 2A–C). Indeed, the dense fibrillar network of the 2D collagen membranes blocked infiltration of more than 99% of bacteria for any species tested, reaching 99.9997% for *E. coli* (Table 1). Even for *P. aeruginosa*, an important opportunist pathogen found on human skin, the 2D collagen membrane was very efficient, allowing infiltration by only 0.117% (± 0.179%) of the 10^7^ CFU bacteria used in the challenge. Although the fluctuation in bacteria able to infiltrate the collagen for this bacterium might appear large, our results indicate that 99.5–100% of *P. aeruginosa* was retained by the 2D collagen membrane in each experiment. Results of viable counts were confirmed by SEM analysis of the lower surfaces of the 2D collagen membranes, on which no bacteria could be detected (Figure 2D–F).

### 2.4. 3D Scaffold Production, Characterization and Mechanical Stability in Wet Conditions

**Figure 3:**
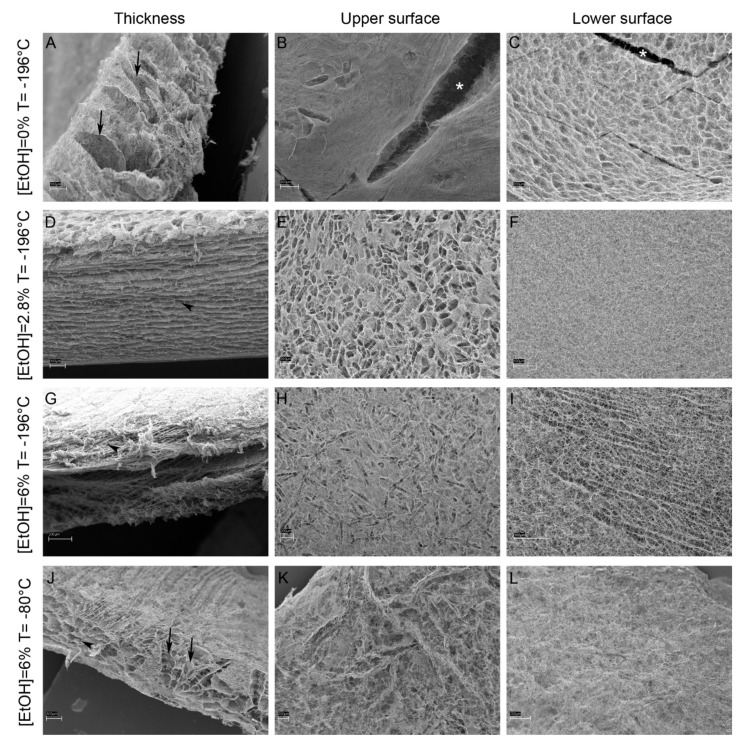
SEM micrographs of 3D scaffolds ([collagen] = 6 mg/mL) with different ethanol concentrations (0%, 2.8% and 6%) and freezing temperatures (−196 °C and −80 °C). First column: 3D scaffold thickness. Second column: 3D scaffold upper surface. Third column: 3D scaffold lower surface. Arrows: vertical channels; arrowheads: horizontal laminae; asterisks: scaffold macroscopic ruptures. Scale bars: **A**,**D**,**F**,**I** = 100 µm; **B**,**C**,**E**,**G**,**H**,**K**,**L** = 200 µm; **J** = 300 µm.

Different parameters, namely fibrillar collagen concentration (4 or 6 mg/mL), additive (ethanol) concentration (2.8%, 6% and 9%) and freezing temperature (−196 °C and −80 °C), were tested to obtain the 3D scaffold with the best performances in terms of microstructural and mechanical features, namely 3D scaffold integrity, presence of laminae and channels within the 3D scaffold thickness, as well as homogeneity and high collagen density of upper and lower surfaces. Increasing ethanol concentrations and different freezing temperatures were pivotal to improve the structure and mechanical resistance of the 3D scaffold (see below). Indeed, all the 3D scaffolds produced in the absence of ethanol displayed macroscopic crevices on the surface, which, on the contrary, were never present in 3D scaffolds with ethanol addition, regardless of its concentration (Figure 3 and Figure S1). At −196 °C and without ethanol, besides macroscopic ruptures (Figure 3B,C), an inner structure based on loose vertical laminae was visible (Figure 3A), whereas dense horizontal laminae were detectable also with the lower ethanol concentrations (2.8% and 6%) (Figure 3D,G). The freezing temperature affected the porosity and pore disposition within the 3D scaffold. Indeed, the simultaneous presence of both vertical channels and horizontal laminae detectable at −80 °C was not visible at −196 °C, considering the same collagen and ethanol concentrations (Figure 3). Both upper and lower surfaces showed differences in terms of superficial porosity and fibrillar collagen density at the same freezing temperature and collagen concentration and at increasing ethanol concentrations (Figure 3). Lower surfaces (Figure 3C,F,I,L) were generally denser than upper surfaces, which showed bigger porosities of petaloid shape (Figure 3B,E,H,K). Homogeneous and dense fibrillar surfaces were visible at both surfaces only at −80 °C (Figure 3K,L). For a summary of the 3D scaffold microstructure depending on the different protocol parameters, see also Table S1.

The mechanical stability in wet conditions was tested soaking the different types of 3D scaffolds in distilled water to simulate the physiological situation of the biomaterial in the cell culture medium (in vitro) or in the wound microenvironment (in vivo). The 3D scaffolds produced at −80 °C (freezing temperature) and with 6% of ethanol concentration were less subjected to structural collapse and deformation, maintaining the original size, shape and thickness to a larger extent than those produced at the same freezing temperature but with 2.8% of ethanol. Figure S2 shows examples of both mechanically stable and unsTable 3D scaffolds.

Overall, considering the data collected from all analyses performed, the best parameters for 3D scaffold microstructure and mechanical stability in wet conditions resulted: 6 mg/mL collagen concentration, 6% ethanol concentration and freezing temperature of −80 °C. Therefore, these parameters were used to produce all 3D scaffolds employed in the subsequent in vitro tests (see below) to ensure coherent and comparable results.

### 2.5. In Vitro Tests

#### 2.5.1. Cytotoxicity

**Figure 4:**
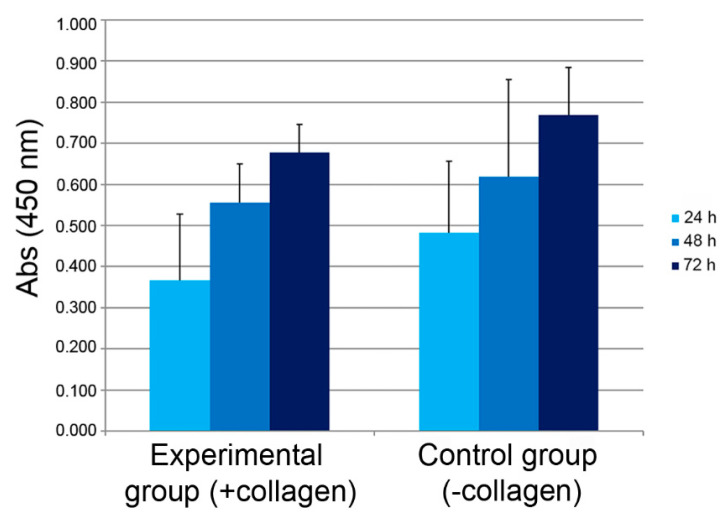
XTT assay. Cells cultured with and without sea urchin-derived collagen progressively proliferate from 24 to 72 h. Differences are not statistically significant (*p* > 0.05). Values: mean ± standard deviation (SD).

The XTT assay performed on short-term hamster fibroblast cultures (24, 48 and 72 h) revealed that the proliferation rate increased in time in both the experimental (cells plus collagen suspension) and control (cells in culture medium only) groups and no statistically significant differences were detectable among groups (*p* > 0.05) (Figure 4).

#### 2.5.2. Evaluation of Cell Infiltration within the 3D Scaffold

**Figure 5:**
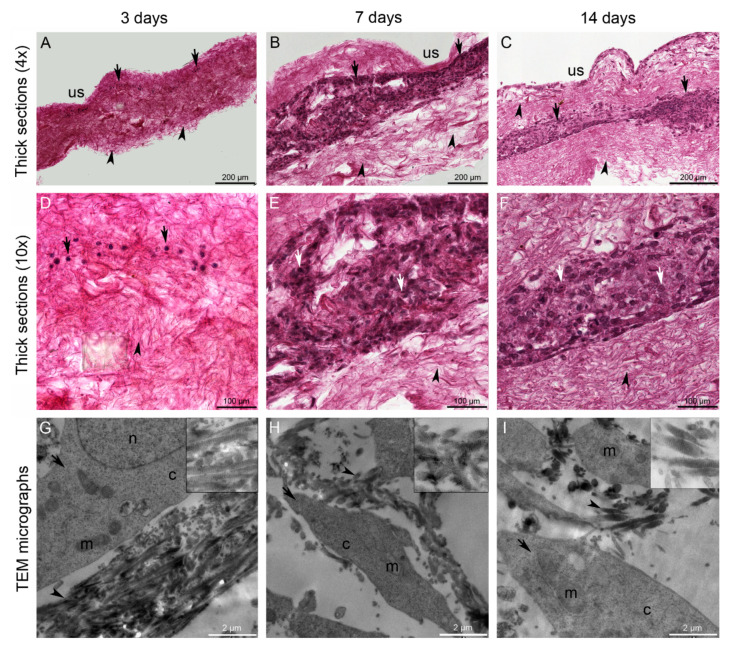
Cells within the 3D scaffolds at short-, medium-, and long-term period (**A**,**D**,**G**: 3 days; **B**,**E**,**H**: 7 days; **C**,**F**,**I**: 14 days). Thick paraffin sections and haematoxylin/eosin staining (**A**–**C** 4x; **D**–**F** 10x), and transmission electron microscopy (TEM) micrographs (**G**–**I**). Fibroblasts easily infiltrate and migrate within the porous 3D scaffolds and directly contact collagen fibrils *via* cell processes. Arrows: cells; arrowheads: collagen fibrils. *Abbreviations*: c: cytoplasm; m: mitochondrion; n: nucleus; us: 3D scaffold upper surface. Top-right inserts in **G**, **H** and **I** show details of collagen fibril ultrastructure, namely the D-period.

Short- (3 days), medium- (7 days) and long-term (14 days) cell cultures were used to evaluate cell infiltration within the 3D scaffold. At each time point, fibroblasts were present not only on the surface of the 3D scaffold but also in the inner portion, indicating that cells could easily infiltrate/migrate within the 3D collagen network (Figure 5). In particular, the infiltration was progressive, as visible from thick sections (Figure 5A–C). At 3 days, cells were spread immediately below the surface of the 3D scaffold, where they were seeded (Figure 5A,D). At 7 days, fibroblasts were also visible deeper in the collagen network (Figure 5B,E). At 14 days, almost all the 3D scaffold area was colonized by cells (Figure 5C,F). Fibroblasts directly contacted the collagen fibrils, easily recognizable by the D-period, by means of cell processes, as revealed by TEM analysis (Figure 5G–I).

#### 2.5.3. Evaluation of Cell Viability and Proliferation within the 3D Scaffold

**Figure 6:**
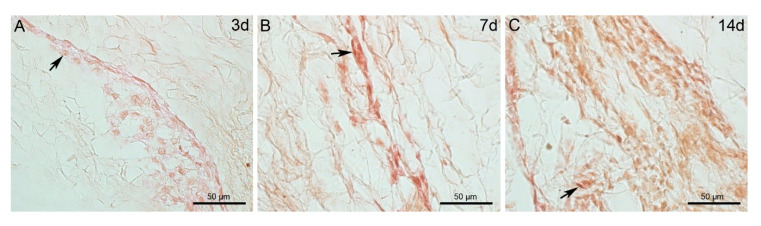
Viability and proliferation of fibroblasts within the 3D scaffolds at short-, medium-, and long-term period using the KI67 marker on thick paraffin sections. **A**: 3 days; **B**: 7 days; **C**: 14 days. At all time-points, proliferating cells are widespread in the 3D scaffolds. *Abbreviations*: d: days. Arrows: KI67-positive cells.

Viability and proliferation of cells infiltrated in the 3D scaffold at the three selected time-points were qualitatively evaluated with the cell proliferation marker KI67. Fibroblasts positive to the proliferation marker were present at 3, 7 and 14 days and were spread in the scaffold (Figure 6), indicating that cells were viable and proliferated from short to long-term periods, an important parameter for future in vivo experiments. The preliminary quantitative analysis performed on a selected slide for each time-point to evaluate fibroblast proliferation rate showed that at 3 days (short-term) KI67-positive cells were 14.2 ± 4.21 (SD), at 7 days (medium-term) 40.2 ± 26.83 (SD), and at 14 days (long-term) 115 ± 19.46 (SD). Overall, KI67-positive cells increased in number from short- to long-term period, confirming the results obtained from microscopy observations (Figure 6).

## 3. Discussion

Collagen is one of the most used biomaterials to produce a wide variety of regenerative medicine tools [31] and shows great advantages as, since it is the main structural protein of the extracellular matrix (ECM), it accurately mimics the natural tissue microenvironment [7]. Collagen-based biomaterials have been used from the XIX century for sutures until now for tissue engineering, regenerative medicine and many other applications [32,33]. 

Skin wounds are nowadays a very significant healthcare problem and collagen-based biomaterials can play an important role in efficiently facing them [5]. The development of skin-like scaffolds which mimic as much as possible functional skin is currently largely investigated [6,34]. Moreover, the opportunity to use collagen sources alternative to mammals is highly relevant in the modern approach of the “green/blue biotechnologies”. Collagen of marine origin is one of the most promising in this respect [14,15,16,26,27]. In this work, we evaluated the potential of sea urchin-derived collagen 2D membranes and 3D scaffolds to produce innovative marine collagen-based skin-like scaffolds (CBSS) for regenerative medicine. 

Collagen extraction yield from the sea urchin peristomial membranes is quite comparable to that already observed for other invertebrates and vertebrates (Table 2). However, it should be considered that, in most cases where higher yields can be obtained, collagen is extracted by hydrolysis, a method that destroys collagen native conformation and therefore its structural-mechanical properties. Thus, slightly higher extraction yield compared to that of sea urchins would imply a lower quality of the material itself, therefore reducing the biomaterial performances and limiting its application potential. Overall, sea urchin-derived collagen may be a valid alternative not only to mammalian-derived collagen but also to other marine-derived collagens, since the extraction yield is comparable and the native conformation of the biomaterial itself is preserved.

As mentioned above, the ideal collagen-based skin-like scaffolds (CBSS) used to heal wounds should be constituted by two layers that mimic the skin structure/function: the epidermis-like and the dermis-like layers. The protocol to produce sea urchin-derived 2D collagen membranes has been previously optimized [26,27] resulting in CBSS possessing good cytocompatibility in vitro [26,27]. In biomedical tools, such as CBSS, the 2D collagen membranes must be tested to evaluate some fundamental epidermis characteristics in case of injury, such as barrier functions. Specifically, the protection from water evaporation and protein loss ensures wound moisture and protein maintenance, important for most skin injury types. The 2D collagen membranes prove to be effective in avoiding/remarkably reducing both water evaporation and protein loss. Since pathogen contamination is one of the major issues in skin wound care, we used both gram-positive and gram-negative bacteria species, different in terms of shape and size, including *S. aureus*, an opportunistic pathogen which is a normal component of the human skin-associated microbiota [43], and *P. aeruginosa*, which represents the main responsible for severe burn-associated skin infections [44] commonly present in clinical injuries. This allowed us to test the natural variability of the microbiota typical of skin wounds. The 2D collagen membrane efficiency in preventing more than 99% of bacterial infiltration after 48 h of incubation here reported is a plus that is often missing in other medical tools employed to repair different skin injuries [45,46]. 

Likewise, the high mechanical resistance of the 2D collagen membranes, already demonstrated in previous works [26,27,47], is an added value since high mechanical resistance is needed in certain applications or in specific injured body parts subjected to mechanical stresses.

The sea urchin-derived 3D collagen scaffolds have been developed to ensure some important dermis features in case of injury, such as cell viability, proliferation, infiltration and migration, to ensure the complete regeneration of the damaged dermal tissues. Our data show that the microstructure (porosity and microstructural arrangement) as well as the mechanical stability of the 3D scaffolds meet all these requirements, since the tested cells are viable, proliferate, and migrate within the scaffold thickness already after few days of seeding. Therefore, these 3D scaffolds show characteristics that are similar to other tools used in regenerative medicine and already tested in vivo in rats [48,49]. Indeed, in terms of microstructure, sea urchin 3D scaffolds present horizontal laminae as seen in scaffolds obtained with collagen extracted with acid-based treatments. Unlike acid-extracted collagen, however, sea urchin 3D scaffolds are formed by native collagen fibrils which may be an advantage in terms of mechanical stability and simulation of a physiological environment. Further, more specific, mechanical tests will be necessary to confirm this hypothesis. 

Another fundamental aspect is the presence, revealed by SEM analysis, of vertical channels (thanks to the addition of ethanol) that seem facilitating cell infiltration and migration within the 3D scaffold. This will be important to evaluate in vivo cell behavior and 3D scaffold suitability for regenerative medicine applications [50]. 

It is noteworthy that the protocol we optimized for the 3D scaffold production can be modified to obtain scaffolds of the desired shape and size, depending on the potential application (different from the CBSS). Indeed, the 3D scaffolds only (without 2D membranes) could be potentially employed for nerve regeneration in tubular conformation, as suggested by [51], for guided bone regeneration, if implemented with hydroxyapatite, as described by [52], to study if cell contraction and migration are influenced by scaffold conformation [50,51], to evaluate cell–ECM interactions [53], growth factors action on cell proliferation or silver nanoparticle antibiotic effects [54].

Sea urchin-derived collagen can be considered cytocompatible. Our present results further support previous data obtained with different cytotypes (horse mesenchymal stromal cells and human skin-derived fibroblasts [26,27]). The cell viability test shows that mammalian fibroblasts seeded in the 3D scaffolds are viable both in short- and long-term cultures. Therefore, sea urchin-derived collagen is promising also for other regenerative medicine applications, as previously suggested [26,27]. Indeed, sea urchin 3D scaffolds could be potentially seeded with suitable cytotypes in vitro (i.e., human fibroblasts) and then implanted in vivo at the level of the wound site, as already suggested for other scaffold types [55].

Overall, sea urchin-derived collagen does not show the main drawbacks of collagen used for skin-like scaffolds reviewed by [56]: indeed, its extraction is not expensive, it is obtainable from an eco-friendly source (i.e., food wastes), and collagen does not lose its native fibrillar conformation and GAG decoration after extraction.

## 4. Materials and Methods

### 4.1. Sea Urchin Fibrillar Collagen Extraction and Extraction Yield

Food industry and restaurant wastes of adult *Paracentrotus lividus* were processed to obtain collagen fibrils in their native conformation as already described [26,27]. Briefly, the peristomial membranes (PM; the soft membrane surrounding the mouth) were isolated from the oral halves of the specimens, singularly weighted, rinsed in artificial sea water, cut in small pieces and left overnight (ON) at room temperature (RT) in hypotonic buffer (10 mM Tris, 0.1% EDTA). After several washes in phosphate buffer saline (PBS), samples were left ON at RT in decellularizing solution (10 mM Tris, 0.1% Sodium Dodecyl Sulphate). Numerous washes in PBS were performed, the disaggregating solution (0.5 M NaCl, 0.1 M Tris-HCl pH 8.0, 0.1 M β-mercaptoethanol, 0.05 M EDTA-Na) was added and left at RT for at least 5 days. All these steps were performed in stirring conditions. The so obtained collagen suspension was then filtered, dialyzed at RT against 0.5 M EDTA for 4 h and against distilled water ON, filtered, aliquoted and stored at −80 °C until use. Collagen concentration (mg/mL) was measured drying and weighting a known aliquot of obtained collagen suspension. The total collagen weight (g) was measured as collagen suspension concentration (mg/mL) × total extracted volume (mL). Then, the extraction yield was calculated as follows: [total collagen weight (g)/total PM fresh weight (g)] × 100.

### 4.2. 2D Collagen Membrane Production

2D collagen membranes were produced using a final collagen concentration of 2 mg/mL, as already described ([27]; see [26,27] for detailed images of produced 2D collagen membranes). UV cross-linking (UV lamp 15 W) was performed overnight at RT. 2D collagen membranes were stored at −20 °C until use (see below).

### 4.3. 2D Collagen Membrane Permeability Tests

**Figure 7:**
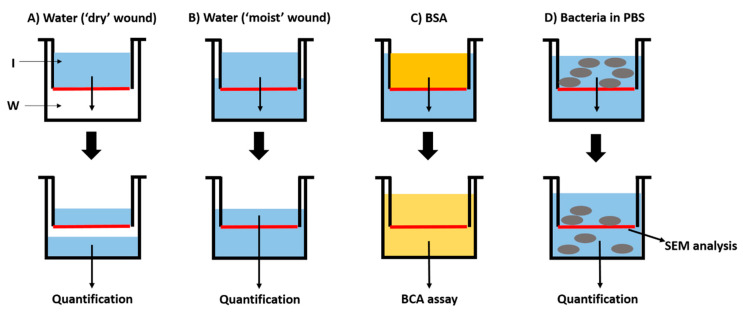
Experimental setup of the 2D collagen membrane permeability tests. **A**: Permeability to distilled water in “dry-wet” conditions (mimicking a “dry” skin wound). **B**: Permeability to distilled water in “wet-wet” conditions (mimicking a “moist” skin wound). **C**: Permeability to proteins (BSA) in “wet-wet” conditions (mimicking a “moist” skin wound). **D**: Bacteria infiltration test. Red lines: 2D collagen membranes. I: insert of the modified Boyden chamber. W: well below the insert of the modified Boyden chamber.

Since the 2D collagen membranes of the CBSS have been developed to mimic the epidermis structure and functions (avoid liquid and protein loss from the wound), they were selected as a suitable form to test permeability to both distilled water and protein, namely bovine serum albumin (BSA). Therefore, 2D collagen membranes were fixed to specific supports (modified Boyden chamber assay) and employed as shown in Figure 7. Experiments were performed at RT with at least two replicates. For distilled water permeability, after preliminary tests performed to validate the effectiveness of the experimental setup, two different experiments were performed to mimic both “dry” (Figure 7A) and “moist” (Figure 7B) skin wounds. For “dry” is meant an injury that is not releasing exudates, whereas for “moist” is meant an injury that is releasing exudates. Therefore, the former can be considered a “dry-wet” situation, where the 2D collagen membrane is in the air at one side and hydrated at the other one, whereas the second can be regarded as a “wet-wet” situation, where both sides of the 2D collagen membrane are hydrated. In the “dry-wet” condition (“dry” wound), 3 mL of distilled water were added in the inserts (I) and water passage through the 2D collagen membranes in the wells below (W) was visually monitored at different time-points, namely 0, 1, 3, 6, 24, 48 h and 7 days (Figure 7A). In the “wet-wet” condition (“moist” wound), at the time-point zero (*t*_0_), 3.5 mL of distilled water were added in the inserts (I) and 2 mL were added in the wells below (W) in order to ensure that both sides of the 2D collagen membranes were hydrated (Figure 7B). In this case, distilled water passage through the 2D collagen membranes was evaluated weighting distilled water inside the inserts at two different time-points, namely 1 day and 7 days (Figure 7B). The percentage of distilled water that passed through the 2D collagen membranes at the previously cited time-points was calculated as follows: [weight (g) of distilled water in the insert at *t*_f_/weight (g) of distilled water in the insert at *t*_0_] × 100, where *t*_f_ and *t*_0_ were the two time-points and the time zero, respectively. Moreover, the mean permeability was also expressed as volume (mL) of distilled water passed through the 2D collagen membrane surface (cm^2^) at both time-points. Since a preliminary test performed in “dry-wet” condition revealed that distilled water did not pass through the 2D collagen membranes, and understood that a soluble protein would not pass through the 2D membranes if its solvent did not pass, for protein permeability, only the “wet-wet” condition (“moist” skin wound) was tested. BSA was prepared in 50 mM PBS (in distilled water, pH 7.4) at a final concentration of 35 mg/mL. 3 mL of BSA were added in the inserts (I) and 3 mL of 50 mM PBS (in distilled water, pH 7.4) in the wells below (W) so that both sides of the 2D collagen membranes were in hydrated conditions (Figure 7C). At each time-point, namely 0, 1, 3, 6, 24, 48 h and 7 days, 100 µL were collected from the wells and BCA protein assay was performed following manufacturer instructions (Thermo Fisher Scientific). Samples were read at the JASCO V-530 Spectrophotometer (wave length: 562 nm). Protein permeability (x) was calculated at each time-point considering the BSA starting concentration (35 mg/mL) as 100%: 35 mg/mL: 100% = [BSA] in the wells at each time-point: x.

### 4.4. Bacteria Infiltration Tests

Since the 2D collagen membranes of the CBSS have been developed to mimic the epidermis structure and functions (avoid entrance of pathogens within the wound), they were selected as a suitable form to evaluate bacteria infiltration. Three bacterial species, namely, the Gram-negative bacilli *Escherichia coli* (MG1655), and *Pseudomonas aeruginosa* (PA01) and the Gram-positive coccus *Staphylococcus aureus* (ATCC29213), were used. 2D collagen membranes were prepared and fixed to specific supports (modified Boyden chamber assay) as described in paragraph 4.3 and shown in Figure 7D. 2 mL of different species of bacteria at a concentration of 10^7^ (5 × 10^6^/mL) in 1× PBS (not growing conditions) were added in the inserts (I) and 2 mL of 1× PBS in the wells below (W) so that the contact between the two solutions would be possible only through the 2D collagen membrane. Modified Boyden chambers were left at 37 °C up to 48 h to analyze bacteria infiltration. Briefly, the 2 mL of 1× PBS inside the wells (W) were collected and centrifuged for 5 min at 5000 rpm to pellet the bacteria that could have passed through the 2D collagen membranes during the 48 h. The pellet was re-suspended in 1 mL of 1× PBS, and 200 µL either of the undiluted sample, or of serial dilutions in 1× PBS (10^−1^ to 10^−5^ dilutions) were plated on LB agar plates and left overnight at 37 °C. Number of colony forming units (CFU) at the various dilutions was determined, and the 2D collagen membrane permeability to bacteria was expressed as percentage (%) of bacteria found in the flow-through (namely, that crossed the 2D collagen membranes) in comparison to the initial bacteria concentration (10^7^ CFU in 2 mL). Consequently, the percentage (%) of retained bacteria was expressed as follows: 100% – % of infiltrated bacteria. To further evaluate bacteria infiltration, both upper and lower surfaces of all 2D collagen membranes were analyzed at the scanning electron microscope (SEM). Briefly, they were fixed overnight at 4 °C in 2% glutaraldehyde in 0.1 M sodium cacodylate tri-hydrate buffer (pH 7.4). After an ON washing in the same buffer at 4 °C, samples were post-fixed in 1% osmium tetroxide in the same buffer for 2 h at RT, washed several times in distilled water, dehydrated in an increasing scale of ethanol and then in solutions of absolute ethanol and hexamethyldisilazane (HMDS) in different proportions (3:1, 1:1, 1:3 and 100% HMDS). Samples were then mounted on suitable stubs, covered with a layer of pure gold (Sputter Coater Nanotech, Assing S.p.A., Rome, Italy) and observed under a scanning electron microscope (LEO 1430, Zeiss, Oberkochen, Germany).

### 4.5. 3D Scaffold Production

The optimization of a protocol for the production of the 3D scaffolds was inspired by [48] to obtain a 3D meshwork of collagen fibrils resembling dermis ultrastructure. Combinations of different parameters were tested: fibrillar collagen suspension concentration (4 mg/mL or 6 mg/mL), freezing temperature (−196 °C or −80 °C), ethanol concentration (2.8%, 6%, 9%). Briefly, fibrillar collagen suspension at a known concentration was centrifuged at 1000 g for 10 min and re-suspended in additive at different concentrations to obtain a final concentration of 4 or 6 mg/mL. Then, the suspensions were added to silicone rubber moulds and immediately frozen at −196 °C (liquid nitrogen) or −80 °C. After lyophilization (Edwards Pirani 1001) ON, UV cross-linking (UV lamp 15 W) was performed ON at RT and scaffolds were stored at −20 °C until use.

### 4.6. 3D Scaffold Characterization

Morphological and microstructural analyses were performed by both light and scanning electron microscopy. Firstly, scaffolds were observed under a LEICA MZ75 stereomicroscope provided with a Leica EC3 Camera and Leica Application Suite LAS EZ Software (Version 1.8.0, Leica Microsystems, Wetzlar, Germany). Secondly, they were mounted on stubs, covered with a layer of pure gold (Sputter Coater Nanotech, Assing S.p.A., Rome, Italy) and observed under a scanning electron microscope (LEO 1430, Zeiss, Oberkochen, Germany). Upper and lower surfaces as well as scaffold thicknesses were analyzed.

### 4.7. 3D Scaffold Mechanical Stability in Wet Conditions

To qualitatively evaluate 3D scaffold stability and therefore select the best parameters to produce a mechanically sTable 3D scaffold in wet conditions, the different 3D scaffold types were submerged in distilled water and the collapse of the 3D scaffolds, namely an evident change in thickness at the naked eye, as well as the resistance to deformation in overall shape and structure were visually evaluated and monitored (visual inspection) immediately after immersion and during the following 24 h under a LEICA MZ75 stereomicroscope provided with a Leica EC3 Camera and Leica Application Suite LAS EZ Software (Version 1.8.0, Leica Microsystems, Wetzlar, Germany). Changes in thickness and overall shape and size were considered as the main qualitative parameters to select the most mechanically stable type of 3D scaffold.

### 4.8. Cell Culture

Hamster fibroblasts (V79) were cultured in DMEM proliferating medium supplemented with 10% (v/v) fetal calf serum (FCS), 100 units/mL penicillin and 100 μg/mL streptomycin on tissue flasks and grown approximately to 80–90% confluence, in a humidified atmosphere of 5% CO_2_ at 37 °C. After 3 days in the proliferating medium, they were then used for the different tests.

### 4.9. In Vitro Tests

#### 4.9.1. Cytotoxicity

This in vitro test was performed to evaluate the viability of hamster fibroblasts when in direct contact with sea urchin-derived fibrillar collagen in suspension. Indeed, fibrillar collagen was re-suspended in cell culture medium to reach a final concentration of 2 mg/mL. In each well of a 96× multiwell plate, 8000 cells were placed as follows in triplicate: cells in cell culture medium only and cells in cell culture + collagen (2 mg/mL). Cell culture medium and 2 mg/mL collagen were respectively used as blanks. The test was performed at 3 time-points: 24, 48 and 72 h. The cell culture medium was removed and cell culture medium without phenol red was added. The colorimetric assay XTT (Cell Proliferation Kit II, Sigma, St. Louis, MO, USA) was performed following kit instructions and 96× multiwells plate was read at a spectrophotometer (Packard-SpectraCount, Meriden, CT, USA) at two wave lengths: 450 and 650 nm. Data were expressed as mean ± standard deviation (SD). Normality was confirmed by a Kolmogorov–Smirnov test (α = 5%). A *t*-test (SPSS 1.1 Version Software, IBM, Endicott, NY, USA) was performed. Differences were considered significant at the *p* ≤ 0.05 level.

#### 4.9.2. Evaluation of Cell Infiltration within the 3D Scaffold

Since the 3D scaffolds of the CBSS have been developed to mimic the dermis and its physiological functions, they were selected as a suitable form to evaluate cell infiltration within a 3D collagen network, simulating the microenvironment that cells have to colonize when the skin is regenerating after an injury. In 24-wells plate, a 50 µL drop of cell culture medium containing 3 × 10^5^ fibroblasts V79 was placed on 3D scaffolds and incubated at 37 °C at 5% CO_2_ for 2 h to let cells infiltrate within the scaffold meshwork. After 2 h, 1 mL of cell culture medium was added in each well. Cell cultures lasted 3, 7 and 14 days and the medium was replaced every 3 days. Five scaffolds were used for each time-point. Three samples per time-point were then processed for classical histological analyses and two samples for transmission electron microscopy analyses (TEM).

For histological analysis, samples were washed with 1× PBS to remove cell culture medium and fixed in 4% paraformaldehyde (PFA) in 1× PBS (pH 7.4) ON at 4 °C. They were then washed in distilled water, dehydrated through a graded ethanol series using an automatic processor (Shandon —Citadel 1000, Thermo Scientific, Waltham, MA, USA) prior to the treatment with xylene and the final embedding in paraffin wax. Samples were then sectioned using a microtome (Leica—RM2035, Leica Microsystems, Wetzlar, Germany) and sections (5–7 µm in thickness) were stained with Harris haematoxylin and eosin. Slides were observed under a Jenaval light microscope provided with a Leica EC3 Camera and Leica Application Suite LAS EZ Software (Version 1.8.0, Leica Microsystems, Wetzlar, Germany).

For TEM analysis, samples were fixed for few days at 4 °C in 2% glutaraldehyde in 0.1 M cacodylate tri-hydrate buffer (pH 7.4) and washed ON at 4 °C in the same buffer. Then, samples were post-fixed in 1% osmium tetroxide in 0.1 M cacodylate tri-hydrate buffer (pH 7.4) for 1 h at RT, washed several times with distilled water and dehydrated in an increasing scale of ethanol. After few washes in propylene oxide, samples were embedded in Araldite 812 epoxy resin. They were sectioned with an ultramicrotome (Reichert Jung Ultracut E, Leica Microsystems, Wetzlar, Germany) and glass knives to obtain ultrathin (70–80 nm) sections that were placed on 300 mesh copper grids, stained with 1% uranyl acetate and lead citrate and observed under a transmission electron microscope (TEM LEO 912AB, Zeiss, Oberkochen, Germany).

#### 4.9.3. Evaluation of Cell Viability and Proliferation within the 3D Scaffold

For the same reasons explained in the previous paragraph, 3D scaffolds were selected as a suitable form to qualitatively evaluate cell viability and proliferation. Therefore, immunohistochemistry analysis with the proliferation marker KI67 was performed to label proliferating fibroblasts within a 3D collagen network. Selected sections of samples embedded in paraffin wax for each time-point (3, 7 and 14 days) were used. Samples were treated with 3 washes in xylene (5 min each), rehydrated in a decreasing scale of ethanol and washed with distilled water and PBS. After treatment with 0.3% Triton X-100 in PBS for 10 min and a wash of 5 min in PBS, samples were treated with 1:20 solution of normal goat serum (NGS) and PBS. A wash in PBS was performed and then they were incubated overnight at 4 °C with the primary mouse monoclonal antibody KI67 (1:100). No primary antibody was added on control samples. Then, samples were washed 3 times in PBS (15 min each) and incubated for 1 h at RT with the anti-mouse secondary antibody (1:200). Few washes in PBS were performed before using the ABC Vectastain Elite (Vector Laboratories; A and B solutions) to amplify the signal. After washing in PBS, the immunoreactive sites were visualized using DAB (3,3-diaminobenzidine tetrahydrochloride) substrate kit (Vector Laboratories). Sections were dehydrated and mounted with Eukitt^®^ (Sigma-Aldrich, St. Louis, MO, USA) and observed under a light microscope Olympus Vanox AHBT3. Moreover, five areas (at 20× magnification) were randomly selected from one slide for each time-point (3, 7, and 14 days) and KI67-positive cells were counted in order to have preliminary quantitative results on fibroblast proliferation rate. Mean cell number and corresponding standard deviation (SD) were measured for each time-point.

## 5. Conclusions

Both the results of our previous studies [26,27] and of the present work underline that sea urchin fibrillar collagen is a valuable biomaterial for the production of marine-derived collagen-based skin-like scaffolds (CBSS). Together with easy handling, these characteristics make the sea urchin-derived CBSS suitable tools for future regenerative medicine applications, especially in the skin injury care field. Further in vivo tests are obviously necessary to prove CBSS immunogenicity and evaluate skin regeneration efficacy. The biomaterial itself is prone to be exploited for biomedical applications different from skin regeneration, since the fibrillar collagen can be easily tuned at need in terms of final tool size and shape.

Moreover, the origin of this marine collagen is an added value: indeed, sea urchin food wastes proved to be promising eco-friendly sources of high-value by-product in terms of collagen extraction yield and application versatility. Obtaining fibrillar collagen from marine wastes improves both circular economy and waste recycling, and the valorization of food waste management will promote sea urchin economy, market, and aquaculture.

Overall, the study of fibrillar collagen derived from this echinoderm could be inspiring for other researches in the biomedical application field in order to combine efforts from different realities (i.e., researchers, food industries, aquacultures, biomedical engineers, doctors and patients) to obtain high quality collagen from wastes to produce tools with high performances for the medical and tissue engineering markets, especially for skin injury treatment.

## Figures and Tables

**Table 1:** *E. coli*, *P. aeruginosa*, and *S. aureus* infiltration. Bacteria were loaded onto the 2D collagen membranes at 10^7^ CFU, as described in Materials and Methods. The numbers reported represent the actual CFU recovered in the flow-through after a 48 h-incubation (namely, that have crossed the 2D collagen membranes), as determined in Materials and Methods, and the mean of the results obtained (5 independent experiments for *E. coli* and *P. aeruginosa* and 4 independent experiments for *S. aureus*; /=absence of experiment). The percentage (%) of infiltrated bacteria is relative to 10^7^ CFU, i.e., the challenge bacterial concentration used in the experiments, with relative standard deviations (St. dev). The percentage (%) of retained bacteria was calculated as described in Materials and Methods.

	*E. coli*	*P. aeruginosa*	*S. aureus*
1st experiment	0	12200	2250
2nd experiment	130	0	100
3rd experiment	0	46400	0
4th experiment	20	0	0
5th experiment	0	0	/
**Mean**	30	11720	587.5
**% of infiltrated bacteria**	0.00030	0.11720	0.005875
**St. dev (+/−)**	0.0005	0.179	0.0096
**% of retained bacteria**	99.9997	99.8828	99.9941

**Table 2:** Collagen extraction yield from different invertebrate (I) and vertebrate (V) sources.

Animal Source	Collagen Extraction Yield (%)	Reference
*Paracentrotus lividus* (I)	4.93	Present study
*Rhizostoma pulmo* (I)	0.2–1	[35]
*Chrysaora* spp. (I)	9–19	[36]
Different squid species (I)	1–11	[37,38]
*Diodon holocanthus* (V)	4–19	[39]
*Thunnus albacares* (V)	1.07–12.1	[40]
Bovines, pigs, sheep (V)	10–30	[41]
Poultry (V)	12.63–30.04	[42]
*Salmo salar* (V)	19.6	[19]
*Gadus morhua* (V)	10.9	[19]

## Supplementary Materials

The following are available online at https://www.mdpi.com/1660-3397/18/8/414/s1, Figure S1: Stereomicroscope images of 3D scaffolds with different ethanol and collagen concentrations, Table S1: Summary of the 3D scaffold structural features depending on the different protocol parameters, Figure S2: Examples of 3D scaffolds during the mechanical stability test in wet conditions.
