# Supplementary material for: From Food Waste to Innovative Biomaterial: Sea Urchin-Derived Collagen for Applications in Skin Regenerative Medicine
Source: Mar Drugs. 2020 Aug 6;18(8):414. doi: 10.3390/md18080414 (PMC7460064; doi:10.3390/md18080414)
Supplement: Supplementary file 1 [file marinedrugs-18-00414-s001.pdf]

Supplementary Materials

From Food Waste to Innovative Biomaterial: Sea Urchin-Derived Collagen for Applications in Skin Regenerative Medicine

Cinzia Ferrario [1,2,*], Francesco Rusconi [1], Albana Pulaj [1], Raffaella Macchi [3], Paolo Landini [3], Moira Paroni [3], Graziano Colombo [3], Tiziana Martinello [4], Luca Melotti [5], Chiara Gomiero [5], M. Daniela Candia Carnevali [1,6], Francesco Bonasoro [1,6], Marco Patruno [5,*] and Michela Sugni [1,2,6,*]

Supplementary Results

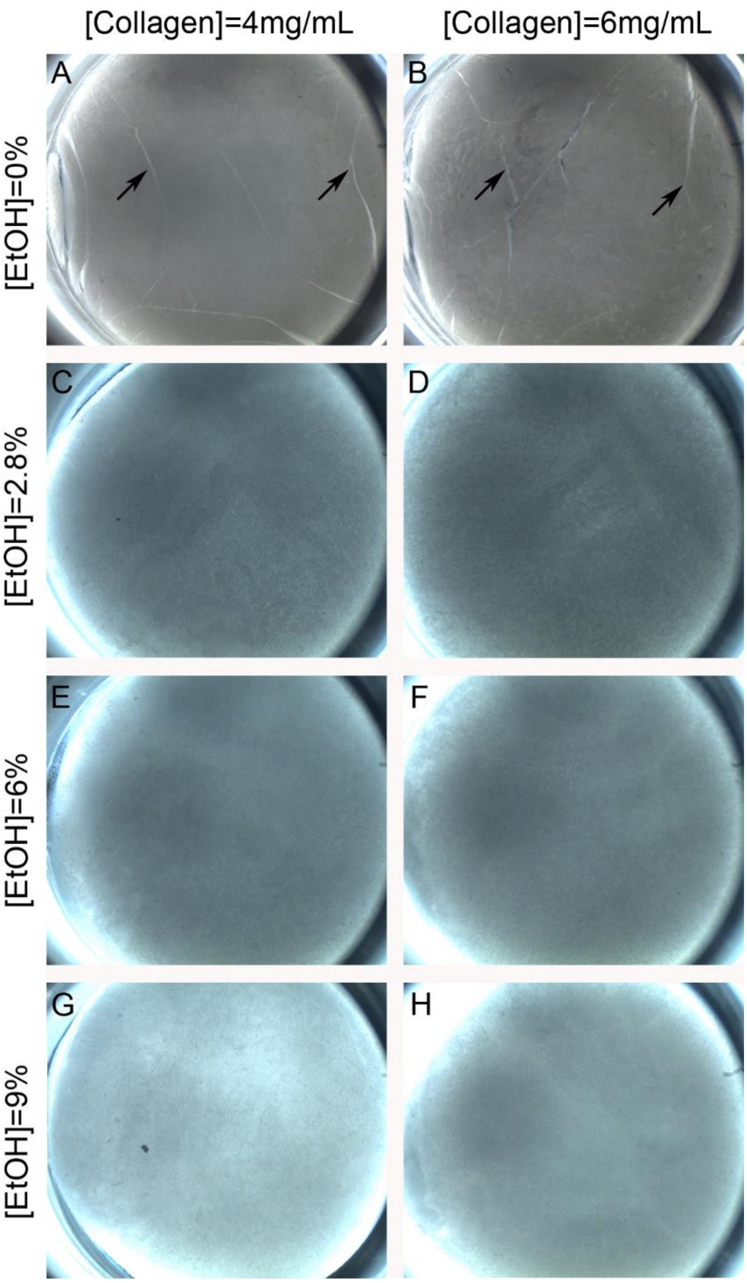

Figure S1. Stereomicroscope images of 3D scaffolds with different ethanol and collagen concentrations. A: 0% EtOH, 4 mg/mL collagen. B: 0% EtOH, 6 mg/mL collagen. C: 2.8% EtOH, 4 mg/mL collagen. D: 2.8% EtOH, 6 mg/mL collagen. E: 6% EtOH, 4 mg/mL collagen. F: 6% EtOH, 6 mg/mL collagen. G: 9% EtOH, 4 mg/mL collagen. H: 9% EtOH, 6 mg/mL collagen. All 3D scaffolds with added ethanol do not show macroscopic ruptures or damages. Arrows: macroscopic ruptures.

Table S1. Summary of the 3D scaffold structural features depending on the different protocol parameters. Collagen concentration was 6 mg/mL for all 3D scaffolds. Features in red were used, together with mechanical stability test results, to select the most suitable parameters, namely [collagen] = 6 mg/mL, [EtOH] = 6%, freezing temperature = -80°C, to produce 3D scaffolds for all subsequent tests.

[EtOH] (%)	Freezing temperature (°C)	Presence of macroscopic ruptures	Presence of vertical channels/ laminae	Presence of horizontal laminae	Simultaneous high collagen density of both upper and lower surfaces
0	-196	Yes	Yes	No	No
2.8	-196	No	No	Yes	No
6	-196	No	No	Yes	No
6	-80	No	Yes	Yes	Yes

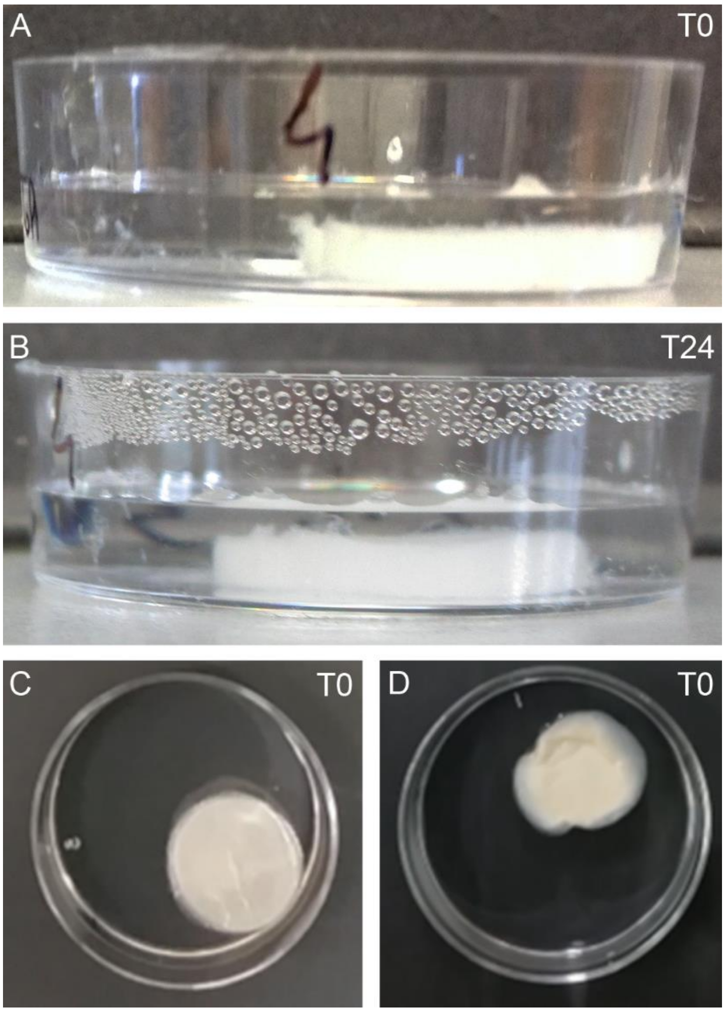

Figure S2. Examples of 3D scaffolds during the mechanical stability test in wet conditions. A: lateral view of a 3D scaffold immediately after immersion in distilled water (T0). B: lateral view of the same 3D scaffold shown in A at 24 hours post-immersion (T24). Thickness of the 3D scaffold does not change after 24 hours of immersion. It can be considered mechanically stable. C: top view of a 3D scaffold immediately after immersion in distilled water (T0). Shape and size are maintained. It can be considered mechanically stable. D: top view of a 3D scaffold immediately after immersion in distilled water (T0). Shape and size are not maintained. It can be considered mechanically unstable. 3D scaffold response to immersion was used to visually and qualitatively evaluate which parameters (collagen and ethanol concentrations, and freezing temperature) would have been more suitable to produce 3D scaffolds for the subsequent tests. Only mechanically stable 3D scaffolds, that do not show changes in thickness (collapse; A and B), and shape and size (deformation; C), were selected.
